# Anuria after kidney transplantation diagnosed as early recurrence of focal segmental glomerulosclerosis combined with acute calcineurin inhibitor nephrotoxicity: a case report and literature review

**DOI:** 10.1186/s12882-024-03524-y

**Published:** 2024-04-05

**Authors:** Yoon-Ju Kim, Seong-Wook Lee, Mee-Seon Kim, Yong-Jin Kim, Ji-Young Choi, Jang-Hee Cho, Chan-Duck Kim, Yong-Lim Kim, Woo-Sung Yun, Seung Huh, Jeong-Hoon Lim, Sun-Hee Park

**Affiliations:** 1 Division of Nephrology, Department of Internal Medicine, School of Medicine, Kyungpook National University, Kyungpook National University Hospital, 130 Dongdeok-ro, Jung-gu, 41944 Daegu, South Korea; 2 Department of Pathology, School of Medicine, Kyungpook National University, Kyungpook National University Hospital, 130 Dongdeok-ro, Jung-gu, 41944 Daegu, South Korea; 3 Department of Surgery, School of Medicine, Kyungpook National University, Kyungpook National University Hospital, 130 Dongdeok-ro, Jung-gu, 41944 Daegu, South Korea

**Keywords:** Anuria, Calcineurin inhibitor toxicity, Focal segmental glomerulosclerosis, Kidney transplantation, Proteinuria

## Abstract

**Background:**

Primary focal segmental glomerulosclerosis (FSGS) is a glomerular disease that sometimes recurs in patients after kidney transplantation (KT) and increases the risk of graft loss. Proteinuria is a common early sign of recurrent FSGS, but an abrupt decrease in urine volume is rare. Herein, we report a patient with early recurrence of FSGS with anuria following KT.

**Case presentation:**

A 55-year-old man with end-stage kidney disease caused by primary FSGS experienced anuria on postoperative day 2 following deceased donor KT. Laboratory results revealed that serum tacrolimus trough levels were consistently elevated at the time of anuria. At first, we considered acute calcineurin inhibitor (CNI) nephrotoxicity based on graft biopsy on light microscopy, laboratory findings, and clinical courses. However, the allograft function did not recover even after discontinuation of CNI, and recurrent FSGS was diagnosed 2 weeks later on electron microscopy. A total of 13 sessions of plasmapheresis and two administrations of rituximab (375 mg/m^2^) were required to treat recurrent FSGS. The patient achieved a partial response, and the spot urine protein-to-creatinine ratio decreased from 15.5 g/g creatinine to 5.2 g/g creatinine. At 5 months following KT, the serum creatinine level was stable at 1.15 mg/dL.

**Conclusions:**

These findings highlight that anuria can occur in cases of early recurrence of FSGS combined with acute CNI nephrotoxicity.

## Background

Primary focal segmental glomerulosclerosis (FSGS) is a common glomerular disease that can lead to end-stage kidney disease [1]. FSGS has a high risk of recurrence after kidney transplantation (KT), with an incidence ranging from 30% after the first KT to 80% after the second KT, if the previous graft was lost due to recurrence of FSGS [2]. In addition, recurrent FSGS can cause poor graft prognosis, such as a rapid decline in graft function and an increase in the risk of graft failure [2].

Podocytes are the major sites of damage in FSGS. They consist of a cell body and multiple foot processes that contact the glomerular basement membrane and form a glomerular filtration barrier to prevent proteinuria [3]. The pathogenesis of FSGS has not yet been clearly elucidated. However, since FSGS mainly recurs early after KT, pre-existing circulating factors, such as soluble urokinase plasminogen activator receptor, cardiotrophin-like cytokine factor-1, apolipoprotein A-lb, and anti-CD40 antibodies, are considered to play important roles in recurrent FSGS [3]. Circulating permeability factors increase glomerular capillary permeability to albumin, which impairs the glomerular filtration barrier and results in persistent proteinuria.

Calcineurin inhibitors (CNIs) are immunosuppressive agents that are commonly used after KT, and they effectively prevent rejection. However, acute CNI nephrotoxicity can occur early after KT. Many studies have demonstrated acute graft dysfunction combined with decreased urine volume related to high serum CNI levels [4, 5]. CNIs are known to cause vasoconstriction of the afferent glomerular arterioles, a reduction in vascular flow, and a decrease in the diameter of afferent arterioles, resulting in nephrotoxicity [6].

Herein, we report a patient who experienced early recurrence of FSGS concurrent with CNI toxicity presenting with anuria after KT. Although several cases of early recurrence of FSGS after KT have been reported, the distinctive feature of this case is the difficult diagnosis of recurrent FSGS with anuria caused by combined acute CNI toxicity.

## Case presentation

A 55-year-old Asian man with end-stage kidney disease caused by primary FSGS, underwent deceased donor KT. At the age of 35, he was diagnosed with primary FSGS and treated with immunosuppressants, including corticosteroids and cyclophosphamide, and later cyclosporin and mycophenolic acid. However, despite treatment, the patient failed to achieve remission. His renal function gradually declined, leading to initiation of hemodialysis by age 44, 9 years after primary FSGS diagnosis. Eleven years after hemodialysis, he was assigned to receive a kidney from a deceased donor. He was in an anuric state and had comorbid hypertension and diabetes. The pretransplant T cell and B cell crossmatch via flow cytometry was negative, respectively without the presence of donor-specific anti-HLA antibodies. The pretransplant panel-reactive antibody values (class I and class II) were 0%. The donor was a 25-year-old woman (height: 161 cm, weight: 70 kg) who was brain-dead and with no comorbidities. The donor had stable vital signs without using vasopressors and had a final serum creatinine level of 0.44 mg/dL, which remained consistently in the normal range throughout the donor management. The kidney donor profile index score was low at 4.

**Fig. 1:**
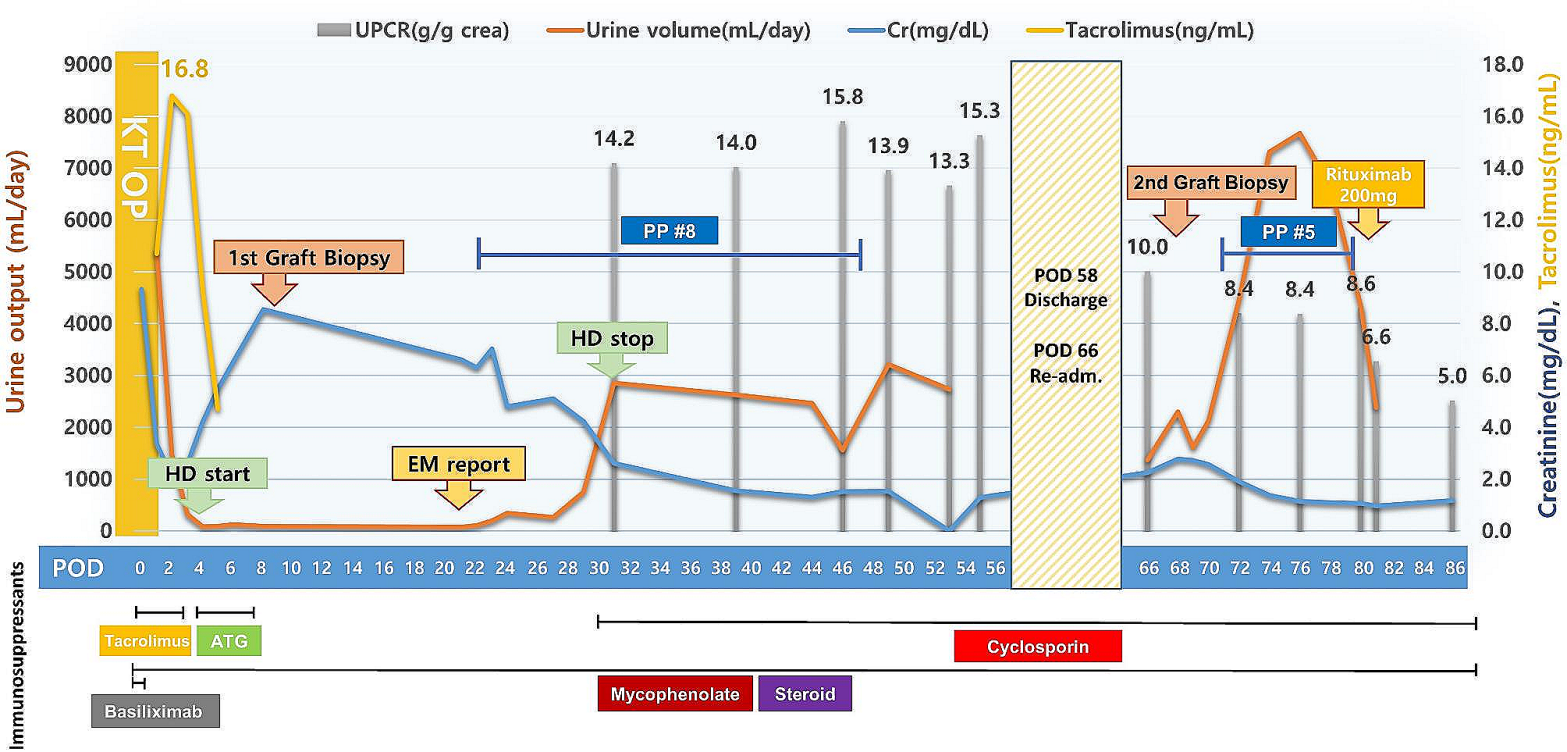
Summary of the clinical course. Abbreviations: UPCR, urine protein-to-creatinine ratio; Cr, creatinine; HD, hemodialysis; EM, electron microscopy; PP, plasmapheresis; ATG, antithymocyte globulin

**Fig. 2:**
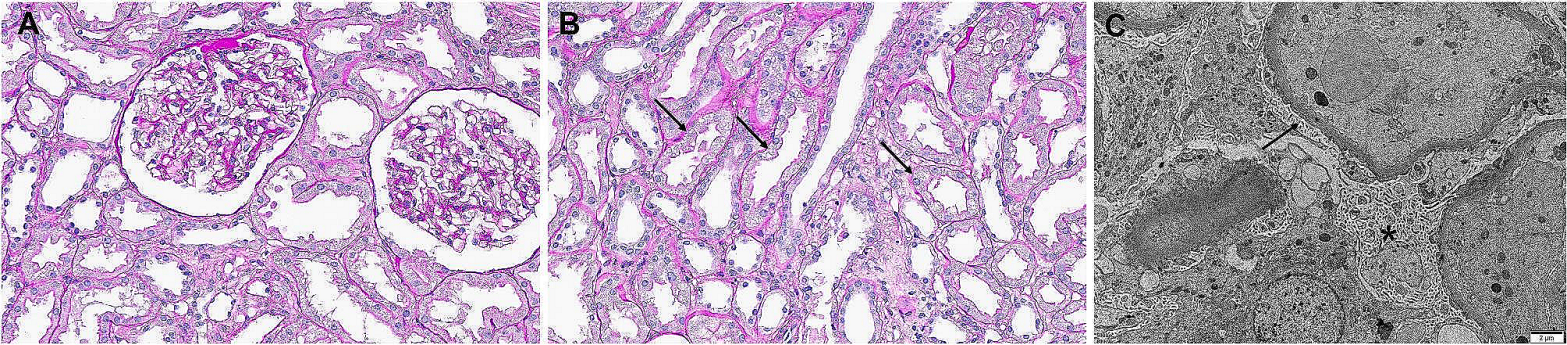
First graft biopsy at 7 days after kidney transplantation. (**A**) The glomeruli have no segmental sclerosis or cellular proliferation. Through the interstitium, inflammatory cell infiltration is absent. (Periodic acid–Schiff [PAS]; original magnification, ×200). (**B**) Isometric vacuolization is noted in the proximal tubular epithelium (arrows). Inflammatory cells are not in the interstitium (PAS; original magnification, ×200). (**C**) Diffuse effacement of foot processes (arrow) along the glomerular basement membrane. Microvilli (*) are formed by the effect of foot process fusion (original magnification, ×5000)

**Fig. 3:**
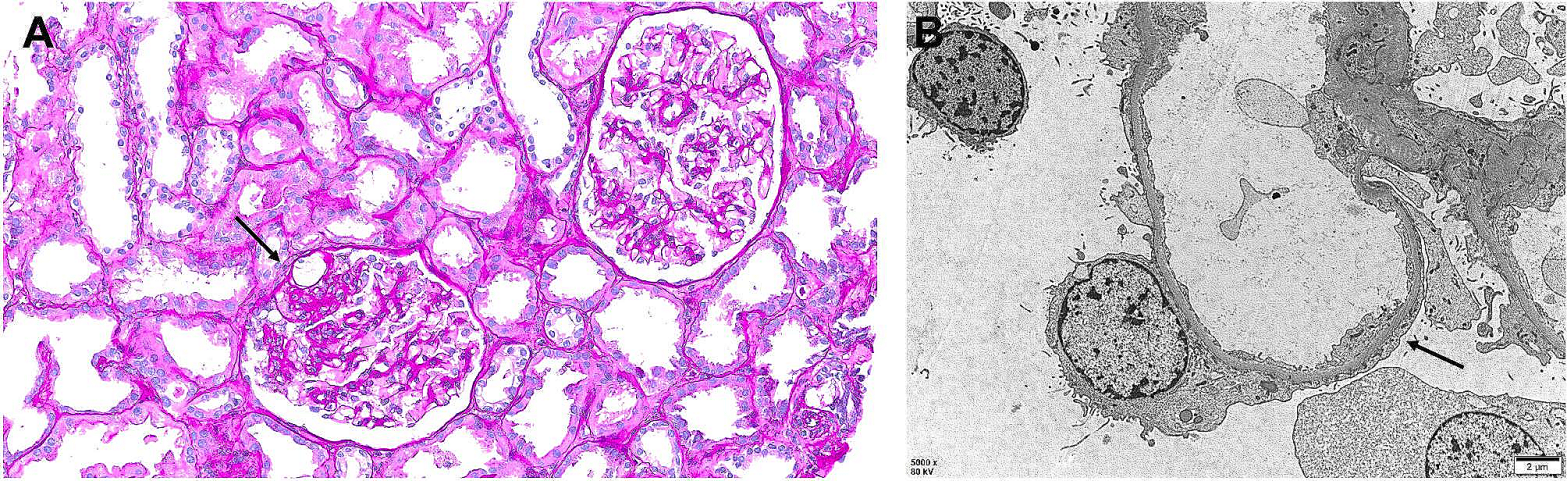
Second graft biopsy at 68 days after kidney transplantation. (**A**) One glomerulus has segmental sclerosis (arrow). The glomerulus at the right upper corner has no sclerotic lesion. Inflammatory cells are not seen in the interstitium (Periodic acid–Schiff [PAS]; original magnification, ×200). (**B**) Diffuse effacement (arrow) of foot processes along the glomerular basement membrane. No electron-dense deposits at the mesangial area (original magnification, ×5000)

During the operation, there were no complications and the patient’s vital signs were maintained within the reference range. The cold ischemic time was 139 min, whereas the warm ischemic time was 41 min. Basiliximab was initially used for induction immunosuppression, and tacrolimus, mycophenolate, and methylprednisolone were used for maintenance immunosuppression. The initial urine volume was good during the first 24 h following KT (6 L/day; Fig. 1). During the 48 h postoperative period, a graft diethylenetriamine pentaacetate (DTPA) scan showed no specific abnormalities, but the urine volume decreased to 1 L/day. Gross hematuria and proteinuria were confirmed with an increase in urine turbidity. Urine analysis revealed the following findings: protein 2+; red blood cells, many/high power field; white blood cells, 3–5/high power field without a urine culture being performed; and increased tacrolimus trough level, 16.8 ng/mL. Subsequently, during the 72-h postoperative period, the urine volume decreased to less than 100 mL/day, and the tacrolimus trough level was still high at 19.6 ng/mL even after a reduced dose of tacrolimus. Moreover, the serum creatinine level gradually increased, and the patient developed a mild tremor in both hands. Based on these findings including clinical course, we considered tacrolimus-induced nephrotoxicity. The graft doppler ultrasound of the graft revealed normal echogenicity without hydronephrosis. The perfusion throughout the graft renal artery, vein, and intragraft was good, and the resistive index was within the normal range. Considering acute tacrolimus nephrotoxicity, tacrolimus was discontinued and antithymocyte globulin was administered. Despite the intervention, anuria continued and the tacrolimus trough level remained elevated at 16.1 ng/mL the next day. Hemodialysis was started on POD 4. The patient’s anuric status did not improve, and a graft biopsy was performed on POD 7. On light microscopy (LM), the glomeruli appeared normal (Fig. 2A), but isometric vacuolization in the tubular cytoplasm was observed, suggesting acute CNI toxicity (Fig. 2B). We found no features of acute tubular necrosis resulting from ischemia, such as tubular epithelial degeneration or detachment from the basement membrane or changes in rejection, either acute T cell-mediated rejection or antibody-mediated rejection. C4d staining confirmed via immunohistochemistry was negative. The Banff scores were all zero. We discontinued tacrolimus, but the anuria persisted. The results of electron microscopy (EM) revealed diffuse foot process effacement suggesting recurrence of FSGS (Fig. 2C). Plasmapheresis for recurrent FSGS was performed for three consecutive days and then once every two days, and the urine volume gradually increased after plasmapheresis initiation. Hemodialysis was discontinued after six sessions of plasmapheresis. The patient was discharged on POD 54 after eight sessions of plasmapheresis. The serum creatinine level was 1.4 mg/dL, but proteinuria did not decrease (15.3 g/g creatinine). After 2 weeks, pitting edema worsened, and the serum creatinine level rose to 2.3 mg/dL. Thus, a second graft biopsy was performed on POD 68. On LM, only one glomerulus had segmental sclerosis out of the 22 glomeruli (Fig. 3A); sclerosis with vacuolization of the overlying podocyte was observed. Lining tubular epithelium of the surrounding tubules were somewhat attenuated, but no epithelial necrosis was noted. No inflammatory cell infiltration along the interstitium; no evidence of rejections. Diffuse foot process effacement on the glomerular basement membrane was seen on EM (Fig. 3B), which were one of the characteristic findings of FSGS. Five additional sessions of plasmapheresis were performed (the patient received a total of 13 sessions of plasmapheresis), and then, rituximab (375 mg/m^2^) was administered twice. The patient remained in partial remission at 5 months after KT, with a serum creatinine level of 1.15 mg/dL and a UPCR of 4.1 g/g creatinine.

## Discussion

We reported the case of a patient with early recurrence of FSGS after KT who developed anuria with combined acute CNI nephrotoxicity. Recurrent FSGS occurs in approximately 30% of patients after KT. However, in this case, the urine volume abruptly decreased to anuria, making it difficult to diagnose recurrence of FSGS. Although proteinuria is an important early finding in cases of FSGS relapse, the occurrence of anuria due to acute CNI nephrotoxicity, as in our case, can make the diagnosis of recurrent FSGS difficult. Thus, these concurrent factors need to be considered to minimize a delay in the diagnosis of recurrent FSGS.

In kidney transplant recipients who had primary FSGS in the native kidney, significant proteinuria of over 1–3 g/g creatinine without other causes of early de novo proteinuria, including acute humoral rejection, was the primary indicator of recurrent FSGS, which ultimately led to a diagnosis through the histological findings of a graft biopsy [7–9]. In the histopathological findings of early recurrence of FSGS, glomeruli usually appear normal on LM, and the diagnosis is confirmed by diffuse foot process effacement on EM [3]. Severe nephrotic range proteinuria in recurrent FSGS can cause graft dysfunction through tubular injury and interstitial edema. However, the incidence of anuria associated with FSGS recurrence is very low. This has been the first case report on the recurrence of FSGS with concurrent anuria, where early recurrence of FSGS combined with acute CNI nephrotoxicity occurred in the graft kidney that had not fully recovered from postoperative ischemic reperfusion injury, resulting in an acute decline in urine volume and long-term anuria.

Of the risk factor for recurrent FSGS, including kidney failure within 3 years of onset, mesangial hypercellularity on initial biopsy, young age at presentation, nephrectomy status, white race, low serum albumin level, and living donor transplantation, this patient had few risk factors [9]. The patient experienced abrupt elevation in creatinine levels, anuria, and hand tremors, which were side effects of increased serum tacrolimus trough levels. Moreover, renal biopsy showed no evidence of FSGS recurrence on LM, which caused a delay in the initial diagnosis of recurrent FSGS until it was eventually confirmed through EM. Although anuria is a common manifestation in CNI toxicity, no previous study has documented its occurrence in recurrent FSGS, leading our group to initially overlook recurrent FSGS as a potential cause of anuria. Therefore, despite the unusual presentation, suspicion of FSGS recurrence is necessary for an early diagnosis of FSGS after KT. Acute tubular necrosis (ATN) needs to be considered as a cause of anuria in the early period following KT. However, in this case, the patient received a kidney from a healthy brain-dead donor with no underlying medical conditions. Additionally, the donor’s renal function was normal, the ischemia time was short, no complications occurred during surgery, and no abnormalities were observed on graft doppler ultrasound. Therefore, based on the temporal relationship and overall clinical course, we initially suspected that the acute graft dysfunction was primarily caused by anuria due to CNI toxicity rather than ATN or postrenal causes.

There are no definite treatment guidelines for recurrent FSGS after KT. However, currently, plasmapheresis with or without rituximab is the most widely used treatment [2]. Plasmapheresis has especially been the mainstay treatment approach for recurrent FSGS. Considering the causal effect of circulating permeability factors for recurrence, it is reasonable to apply plasmapheresis to target circulating factors. In addition to plasmapheresis, rituximab, which is a monoclonal antibody targeting CD20 expressed in B lymphocytes, has been used to treat recurrent FSGS. Despite the beneficial effect of rituximab on FSGS, the pathophysiologic mechanisms of its action are not fully elucidated. The potential mechanism of action of rituximab involves a decrease in the production of circulating permeability factors through the depletion of B lymphocytes, and it also has a direct protective effect on podocytes through the stabilization of proteins expressed on podocytes [10].

Table 1 summarizes the results of studies that evaluated the course and treatment of recurrence of primary FSGS after KT. Uffing et al. reported the outcomes of recurrent FSGS after KT in an international cohort from Europe, Brazil, and the United States [11]. Among 176 patients with primary FSGS, 57 (32%) experienced recurrence of FSGS, with a median time to recurrence of 1.5 months. Patients with recurrent FSGS had a 5-fold higher risk of graft loss than those without recurrence. The authors also evaluated treatment responses according to the types of immunosuppressive treatments. Among patients who received plasmapheresis, the complete remission rate was 28% and the partial remission rate was 44%. When rituximab was added to plasmapheresis, the complete remission rate was 17% and the partial remission rate was 30%. In a French study conducted by Lanaret et al. [2], of 914 patients with primary FSGS who underwent KT, 165 (18%) experienced recurrence of FSGS, with a mean time to recurrence of 7 days. Among them, 148 patients were divided into two groups to evaluate the efficacy of rituximab for treating recurrent FSGS after KT. Group 1 received the standard of care (SOC; plasmapheresis, high dose corticosteroids and high dose CNIs), and some of the patients received rituximab because of the treatment failure of the SOC or the early discontinuation of plasmapheresis after the achievement of remission. Group 2 received the SOC along with early use of rituximab either for recurrence prevention on the day of transplantation or immediately (within 28 days) after recurrence. The remission rates in groups 1 and 2 were 82.6% and 71.8%, respectively, without a significant difference between the two groups. Among nonresponders to the SOC who received rituximab in group 1, 57.9% achieved remission. This finding suggests that rituximab may be beneficial when patients do not respond to conventional treatment. Recently, Kwon et al. reported a single-center retrospective study analyzing South Korean kidney transplant recipients with biopsy-proven primary FSGS [8]. Of 99 patients, 21 (21.2%) experienced recurrence of FSGS, with a median time to recurrence of 6 days. Among the 21 patients with recurrence, 18 (85.7%) were treated with plasmapheresis only and 3 (14.3%) were treated with plasmapheresis and rituximab. The complete remission rate was 39.1%, and the partial remission rate was 47.6%. A single-center retrospective study conducted in the United States reported long-term outcomes of transplant recipients who experienced recurrent FSGS [12]. The recurrence rate was 26% in 100 patients with primary FSGS, with the median time to recurrence being 4.5 days following KT. To treat recurrence, 24 (96%) patients received plasmapheresis, and 11 (46%) received plasmapheresis along with rituximab. Among these patients, 18 (72%) had complete or partial remission after treatment, and the overall graft loss rate following recurrence was 12% over a median period of 1.5 years.

**Table 1:** Summary of studies on FSGS recurrence after kidney transplantation

Study	Study design	Nation	Number of patients	Age at FSGS diagnosis (years)	Age at transplantation (years)	Time to ESKD (months)	Number of recurrent patients, n (%)	Time to relapse of FSGS (days)	Treatment	Treatment response (CR/PR)
**Uffing et al.** [11]	Retrospective	Europe, Brazil, and US	176	27	38	38	57 (32%)	45	PP PP + RTX	CR/PR: 28%/44% CR/PR: 17%/30%
**Lanaret et al.** [2]	Retrospective	France	914	25.4	39.9	68	165 (18%)	7	SOC + late RTX SOC + early RTX	CR/PR: 45.9%/36.7% CR/PR: 48.7%/23.1%
**Kwon et al.** [8]	Retrospective	South Korea	99	-	37.1	70	21 (21%)	6	PP ± RTX	CR/PR: 39.1%/47.6%
**Wood et al.** [12]	Retrospective	United States	100	-	34.3	-	26 (26%)	4.5	PP ± RTX	CR + PR: 72%

Abbreviations: FSGS, focal segmental glomerulosclerosis; ESKD, end-stage kidney disease; CR, complete remission; PR, partial remission; PP, plasmapheresis; RTX, rituximab; SOC, standard of care

Currently, no international guidelines have been established for the use of rituximab in the treatment of recurrent FSGS after KT. Studies conducted thus far have been limited to retrospective analysis. Most studies on the use of rituximab in recurrent FSGS have been conducted on patients who were unresponsive to initial plasmapheresis treatment, those who desired early plasmapheresis discontinuation, or those receiving plasmapheresis as prophylaxis for recurrent FSGS in high-risk populations. Therefore, large-scale prospective studies are needed to confirm the efficacy and safety of rituximab as a treatment for recurrent FSGS in KT recipients.

To date, numerous retrospective analyses have been conducted to understand and potentially prevent the recurrence of FSGS after KT and improve graft outcomes. Kwon et al. evaluated whether recurrent FSGS after KT was reduced by pretransplant plasmapheresis with or without rituximab [8]. It was found that the rate of postoperative recurrence was significantly lower in the pretreatment group than in the non-pretreatment group (9.4% vs. 34.8%; *p* = 0.002). On the other hand, Alasfar et al. assessed the efficacy of plasmapheresis with rituximab for the prevention of recurrence of FSGS [9]. In that study, 66 patients with FSGS from African American (*n* = 21, 32%), Asian (*n* = 5, 7%), and Hispanic (*n* = 3, 4%) backgrounds were enrolled. Among them, 37 patients (56.1%) with a high risk of recurrence received preventive therapy with plasmapheresis and rituximab. Recurrence was noted in 23 (62.2%) patients from the pretreatment group and 14 (48.3%) from the non-pretreatment group, and the rate did not differ between the groups. However, the authors emphasized that early diagnosis and treatment of recurrence may result in improved outcomes. These studies showed different results of the effectiveness of preventive treatment for recurrent FSGS after KT, which may be associated with the differences in racial characteristics. In addition, whether high-risk patients were selectively targeted for preventive treatment may have influenced the results. Therefore, large-scale studies are needed to provide definite guidelines for the prevention and treatment of recurrent FSGS.

## Conclusions

Recurrence of FSGS can occur early after KT with anuria when combined with acute CNI nephrotoxicity. This complicates the early diagnosis of recurrence of FSGS. Therefore, clinicians should keep this possibility in mind and be careful not to delay the diagnosis of recurrent FSGS.

## Data Availability

The data generated and analyzed in this case are presented within the manuscript.

## Abbreviations

FSGS: Focal segmental glomerulosclerosis
KT: Kidney transplantation
CNI: Calcineurin inhibitor
DTPA: Diethylenetriamine pentaacetate
POD: Postoperative day
LM: Light microscopy
EM: Electron microscopy
UPCR: Urine protein-to-creatinine ratio

## References

[1] Bose B, Cattran D (2014). Glomerular diseases: FSGS. Clin J Am Soc Nephrol.

[2] Lanaret C, Anglicheau D, Audard V, Büchler M, Caillard S, Couzi L (2021). Rituximab for recurrence of primary focal segmental glomerulosclerosis after kidney transplantation: results of a nationwide study. Am J Transpl.

[3] Shoji J, Mii A, Terasaki M, Shimizu A (2020). Update on recurrent focal segmental glomerulosclerosis in kidney transplantation. Nephron.

[4] Matas AJ (2011). Chronic progressive calcineurin nephrotoxicity: an overstated concept. Am J Transpl.

[5] Goździk M, Płuciennik A, Zawiasa-Bryszewska A, Nowicka M, Nowicka Z, Wągrowska-Danilewicz M (2019). Acute kidney injury following exposure to calcineurin inhibitors in a patient with idiopathic membranous nephropathy. Drug Saf Case Rep.

[6] Hošková L, Málek I, Kopkan L, Kautzner J (2017). Pathophysiological mechanisms of calcineurin inhibitor-induced nephrotoxicity and arterial hypertension. Physiol Res.

[7] Naciri Bennani H, Elimby L, Terrec F, Malvezzi P, Noble J, Jouve T et al. Kidney transplantation for focal segmental glomerulosclerosis: can we prevent its recurrence? Personal experience and literature review. J Clin Med. 2021;11. 10.3390/jcm11010093 PMC8745094 35011834

[8] Kwon HE, Kim YH, Lee SA, Lee JJ, Ko Y, Shin S (2023). Post-operative recurrence of focal segmental glomerulosclerosis according to pre-transplant treatment after kidney transplantation. BMC Nephrol.

[9] Alasfar S, Matar D, Montgomery RA, Desai N, Lonze B, Vujjini V (2018). Rituximab and therapeutic plasma exchange in recurrent focal segmental glomerulosclerosis postkidney transplantation. Transplantation.

[10] Tedesco M, Mescia F, Pisani I, Allinovi M, Casazza G, Del Vecchio L (2022). The role of Rituximab in primary focal segmental glomerular sclerosis of the adult. Kidney Int Rep.

[11] Uffing A, Pérez-Sáez MJ, Mazzali M, Manfro RC, Bauer AC, de Sottomaior Drumond F (2020). Recurrence of FSGS after kidney transplantation in adults. Clin J Am Soc Nephrol.

[12] Wood EL, Kwan L, Burrows JE, Singh G, Veale J, Lum EL (2023). Early recurrence of focal segmental glomerulosclerosis in kidney transplant recipients: when to consider regifting. Transplantation Rep.

